# Supplementary material for: Seasonal plasticity in anti‐predatory strategies: Matching of color and color preference for effective crypsis
Source: Evol Lett. 2019 Apr 10;3(3):313–20. doi: 10.1002/evl3.113 (PMC6546441; doi:10.1002/evl3.113)
Supplement: Supplementary file 1 — Fig. S1. Schematic representation of the experimental design. Fig. S2. Colour calibration and linearisation of the scanner. Fig. S3. Quantification of crypsis‐enhancing pigmentation traits. Fig. S4. Behavioural data per cohort. Fig. S5. Correlations between eye size and perching preference. [file EVL3-3-313-s001.doc]

# SUPPLEMENTARY MATERIALS

Supplementary Fig. 1 Schematic representation of the experimental design.

Supplementary Fig. 2 Colour calibration and linearisation of the scanner.

Supplementary Fig. 3 Quantification of crypsis-enhancing pigmentation traits.

Supplementary Fig. 4 Behavioural data per cohort.

Supplementary Fig. 5 Correlations between eye size and perching preference.

Supplementary Table 1 Colours and sizes of the wing pattern elements across treatments.

Supplementary Table 2 Generalized linear mixed models for perching preference.

Supplementary Table 3 Generalized linear mixed models for activity levels.

Supplementary Table 4* Behavioural dataset used in this study.

Supplementary Table 5* Morphological dataset used in this study.

* Supplementary Table 4 and 5 will be uploaded to Dryad upon acceptance for publication.

**Supplementary Figure 1. Schematic representation of the experimental design.** Panels on the left-hand side depict the natural environment of *B. anynana* during the wet season (upper) and dry season (lower) while panels on the right-hand side provide a schematic representation of our experimental design. Individuals were reared, from egg to adult eclosion, at one of two developmental temperatures (Td; 19°C and 27°C) that simulate the ambient temperatures of the dry and wet season, respectively. Variation in rearing temperature is sufficient to induce seasonal polymorphism in this species, with cool and warm temperatures inducing dry season form (DS) and wet season form (WS) individuals, respectively. On the day of eclosion, the butterflies were allocated to one of two adult temperatures (Ta; 19°C and 27°C), yielding four experimental treatments with different combinations of Td and Ta. Behavioural assays were performed at the temperature of the adult regime using cohorts of 20 six to eight day old males or females (N_cohort_ = 64). The red colours in this scheme represent warm temperatures or individuals reared at warm temperatures. Cool temperatures, or individuals reared at cool temperatures, are represented by blue colours. Photographs of natural environments are courtesy of Jonas Ardö**.**


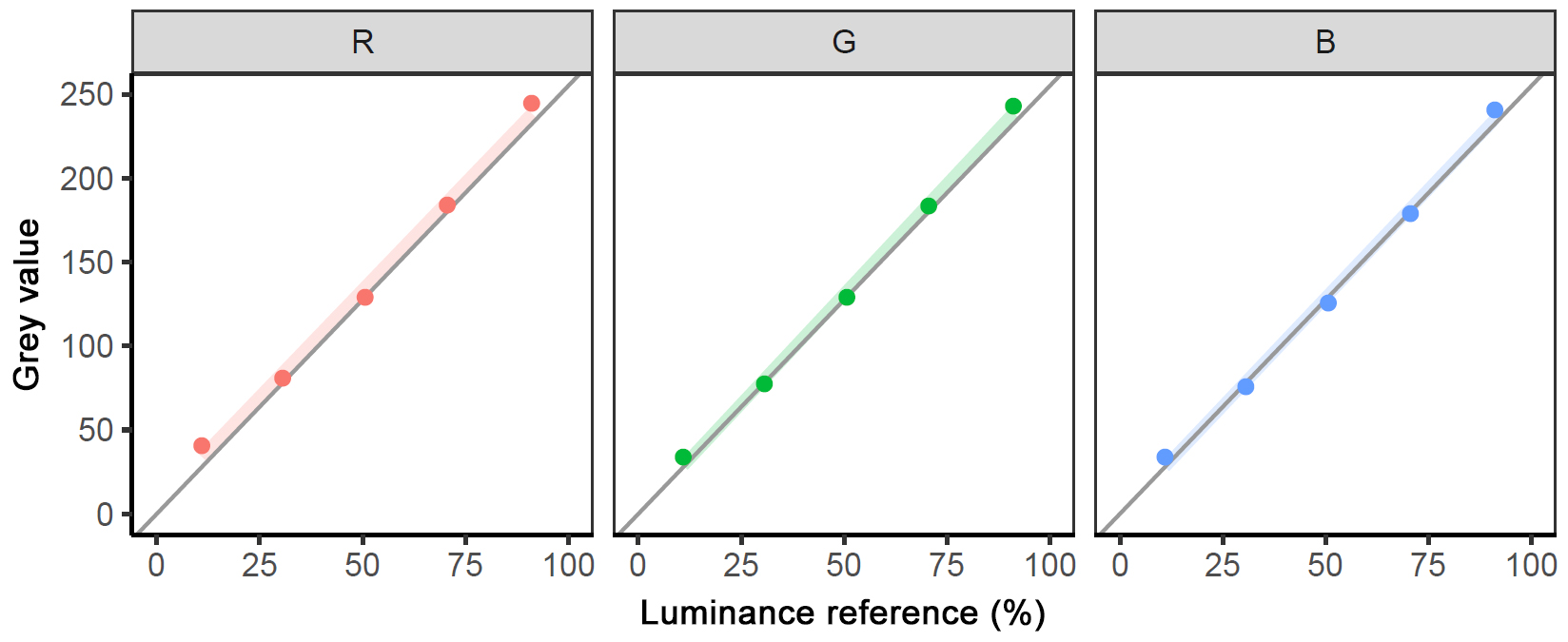
**Supplementary Figure 2. Colour calibration and linearisation** **of the scanner.** The digital scanner (Epson V600) used to acquire wing colour information was calibrated using a colour reference card (Wolf Faust, R110714). The relationship between the grey scale value measured for a set of five reflectance standards (GS0, GS5, GS10, GS15 and G20 on the colour reference card) shows that the gamma curves are linear with a close fit to the required values (grey lines) in each of the three colour channels; R (longwave or ‘red’), G (mediumwave or ‘green’) and B (shortwave or ‘blue’).


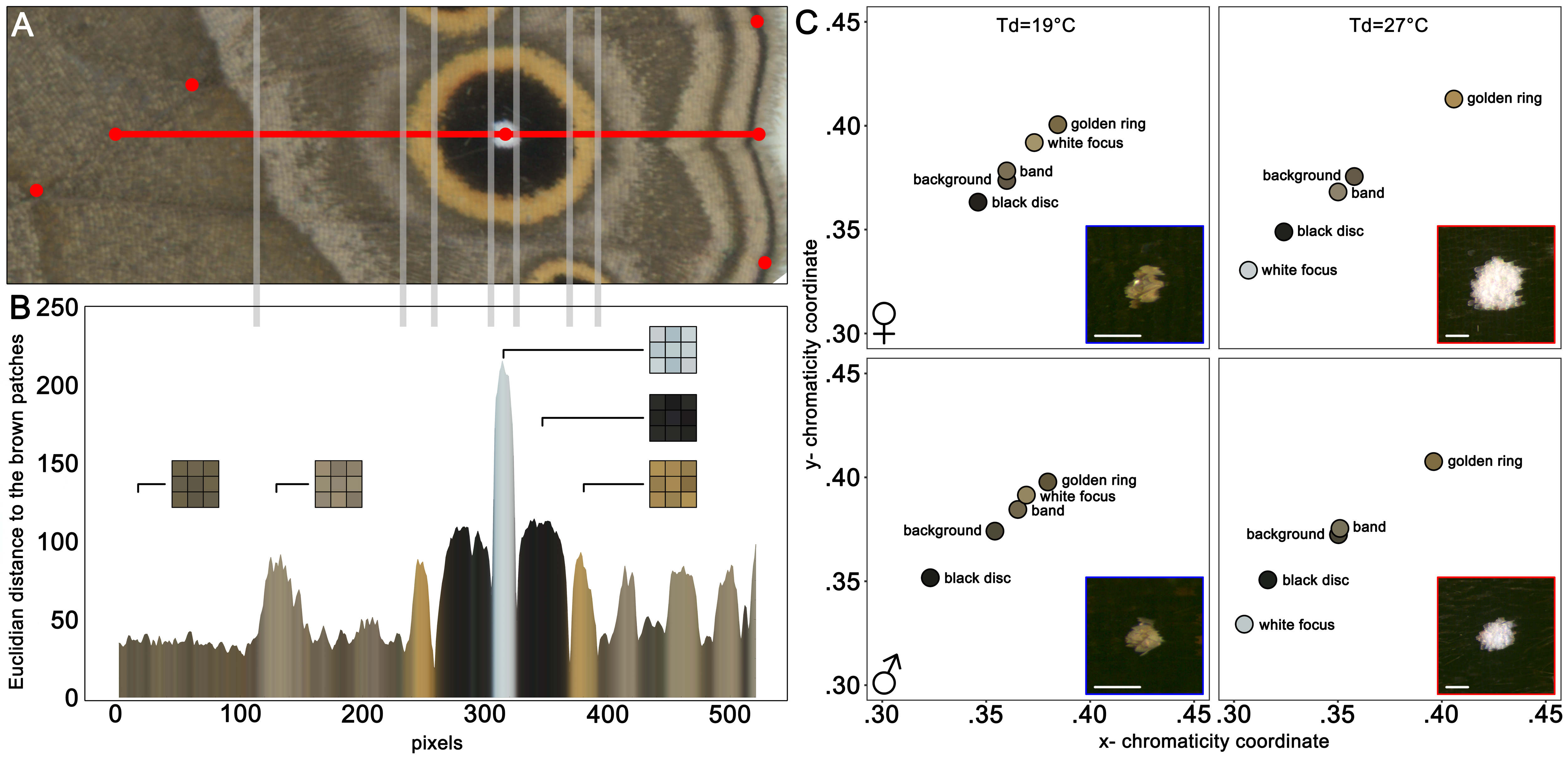
**Supplementary Figure 3. Quantification of crypsis-enhancing pigmentation traits. A.** We used a set of custom-made interactive Mathematica notebooks to analyse the ventral hindwing surfaces of 16 female and 16 male butterflies per experimental treatment (i.e. two individuals per cohort, N=128). Two contiguous transects were defined by the centre of the large fifth eyespot (cell Cu1) and four wing landmarks (on the wing margin and intersection between veins). The four wing landmarks were used to calculate midpoint values along the wing vein and margin, and these midpoints determined the start and end of the contiguous transects. Secondary landmarks, represented by grey lines, were used to specify the edge of the central band and the limits of each of the colour rings along the transect line. **B.** The colours of the eyespot rings were quantified by extracting the mean RGB values of 3×3 pixel squares centred on the intersection between the midpoints of the secondary landmarks and the transect line. The mean RGB values of the background and central band were extracted from a 3×3 cluster located 10 pixels after the associated secondary landmarks on the marginal side. Here, each pixel along the transect is represented as its Euclidean distance, in RGB colour space, to the colour of the brown patches used in our behavioural assays. **C.** RGB values were converted (to CIE-xyY colour space) and represented in CIE-xy chromaticity diagrams (a normalized representation of the colours perceived by trichromatic observers). To estimate how similar or dissimilar the colours of wing pattern elements are we calculated the Euclidean distance between the colour of the each pattern element and the colour of the wing’s background. CIE-xy chromaticity diagrams give the mean values for the each pattern element for females (upper) and males (lower) reared at cool (Td=19°C; left) and warm (Td=27°C; right) temperatures. The inset in each plot represent a detailed image of the wing scales that make up the white focus of the eyespot.


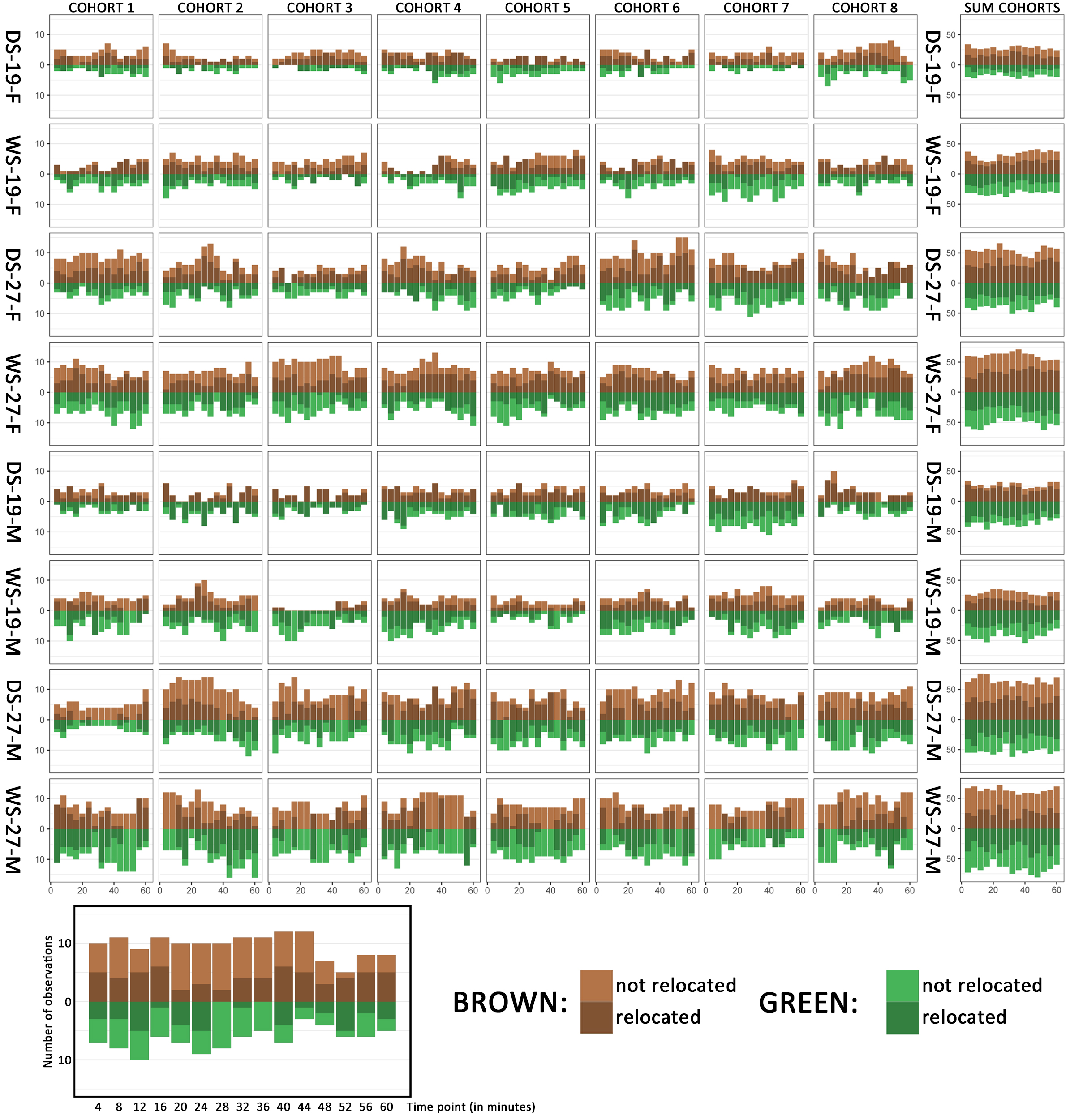


**Supplementary Figure 4. Behavioural data per cohort.** Panels represent all behavioural data per cohort. The number of butterflies at rest on each of the two background colours is represented by the brown and green bars for each time point. The number of individuals resting at exactly the same position as in the previous time point (i.e. no re-location) is depicted by lighter colours inside each bar. Note that individuals that were resting on the mesh netting that covered the front of the cage or on the upper surface of the cage, or were airborne when the time-lapse photos were taken were denoted as ‘unaccounted for’ in the data. The number of individuals that was unaccounted for was higher at cooler temperatures (Ta=19°C) for both males and females.


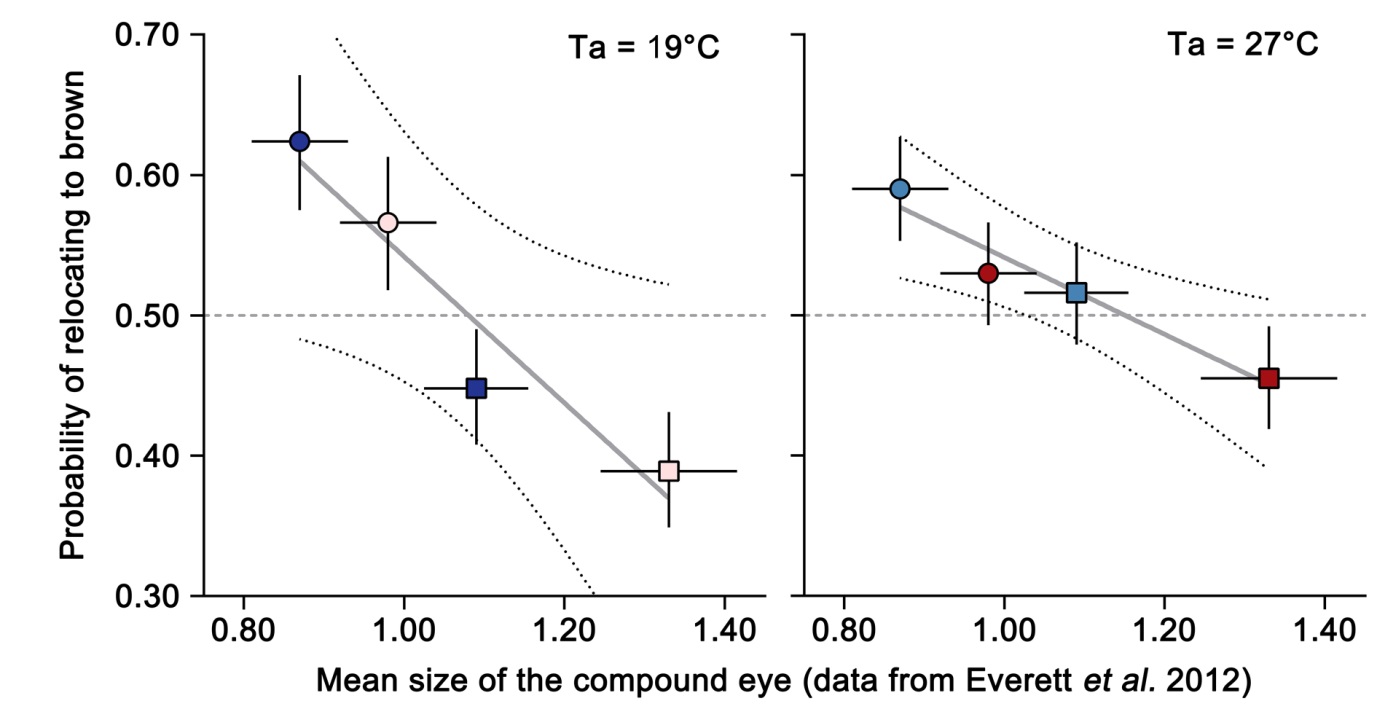


**Supplementary Figure 5. Correlations between eye size and perching preference.** The size of the *B. anynana* compound eye is known to be developmentally plastic and sexually dimorphic. Individuals reared at warm temperatures (wet-season; red dots) have larger eyes than individuals reared at cold temperatures (dry-season; blue dots) and females (circles) have smaller eyes than males (squares); data from Everett *et al.* 2012. Our behavioural data on perching preferences (y-axis) correlates strongly with the published data on thermal plasticity in eye size (x-axis) such that individuals with smaller eyes have stronger preferences for brown patches. Left-hand panel gives the correlation between eye size and perching preferences measured at cool temperatures (Ta=19°C; r=-0.522, R^2^=0.914, P=0.044) while the right-hand panel shows the correlation with behavioural responses assessed at warm temperatures (Ta=27°C; r=-0.274, R^2^=0.949, P=0.026).
