# Supplementary material for: Seasonal plasticity in anti‐predatory strategies: Matching of color and color preference for effective crypsis
Source: Evol Lett. 2019 Apr 10;3(3):313–20. doi: 10.1002/evl3.113 (PMC6546441; doi:10.1002/evl3.113)
Supplement: Supplementary file 2 — Table S1. Colours and sizes of the wing pattern elements across treatments. Table S2. GLMM for perching preference (related to Fig 2a in the main text). Table S3. GLMM for activity levels (related to Fig 2b in the main text). [file EVL3-3-313-s002.doc]

| **Supplementary Table 1.** Colours and sizes of the wing pattern elements across treatments. Left-hand panels represent the Euclidian distance in CIE-xyY colour space from the colour of the brown patches used in the experiment to **A)** all pixels along the transect and **B)** the background colour of the wing. Panels C to F represent the Euclidian distance from the background colour of each individual to **C)** the central band, **D)** the golden ring, **E)** the black disc and **F)** the white focus. Panels G to K are related to (relative) sizes and panels represent **G)** the length of the transect (i.e. proxy for wing size), **H)** the relative distance from the start of the transect to the proximal edge of the central band (i.e. inverse proxy for the width of the band, which is difficult to measure directly because of its indistinct distal edge), **I)** the relative width of the golden ring, **J)** the relative width of the black disc and **K)** the relative width of the white focus. Relative widths were calculated by dividing the absolute size by the length of the transect. Tables on the right-hand side of each panel give the effects of developmental temperature (Td), adult temperature (Ta), sex and their interactions on the phenotypic trait measured. Effects with P<0.01 are shown in bold. The experimental cohort was included as a random factor and denominator degrees of freedom were determined with the Kenward-Roger method, using the package *afex*. Post-hoc pairwise comparisons (alpha=0.05) were conducted using the package *lsmeans*. Differences between experimental treatments and sexes are given in the graphs, with groups that are not significantly different from each other sharing a common letter. | | | | | | |  |  |
| --- | --- | --- | --- | --- | --- | --- | --- | --- |
| **A)** | 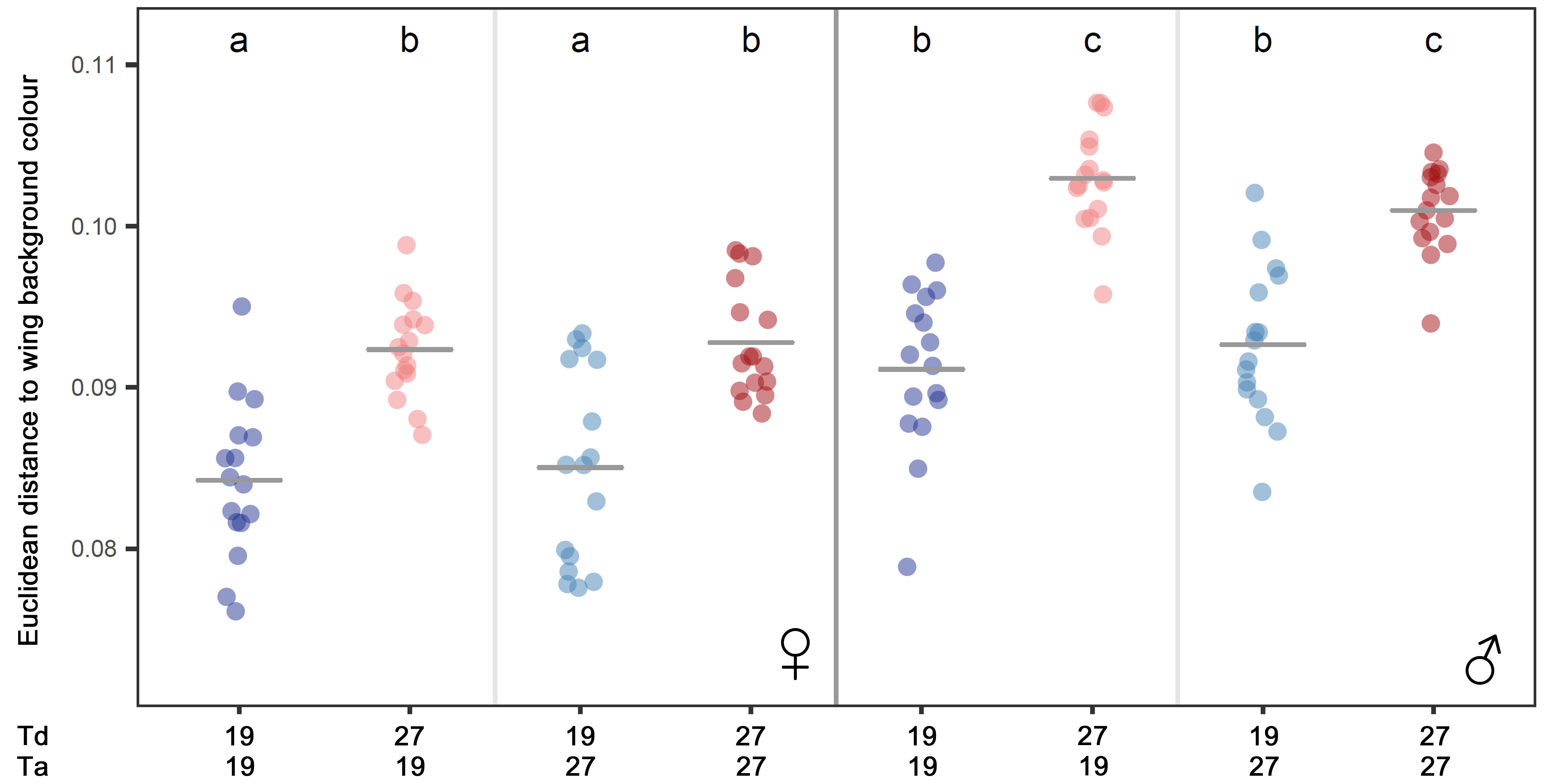 |  | F-value | df_KR_ | P_KR_ | | | |
|  |  | **T_D_** | **134.70** | **1,56** | **<0.001** | | | |
|  |  | T_A_ | 0.06 | 1,56 | 0.806 | | | |
|  |  | **Sex** | **115.02** | **1,56** | **<0.001** | | | |
|  |  | T_D_:T_A_ | 1.52 | 1,56 | 0.222 | | | |
|  |  | T_D_:Sex | 1.93 | 1,56 | 0.170 | | | |
|  |  | T_A_:Sex | 0.29 | 1,56 | 0.590 | | | |
|  |  | T_D_:T_A_:Sex | 1.04 | 1,56 | 0.312 | | | |
|  | | | | | |  |  |  |
| **B)** | 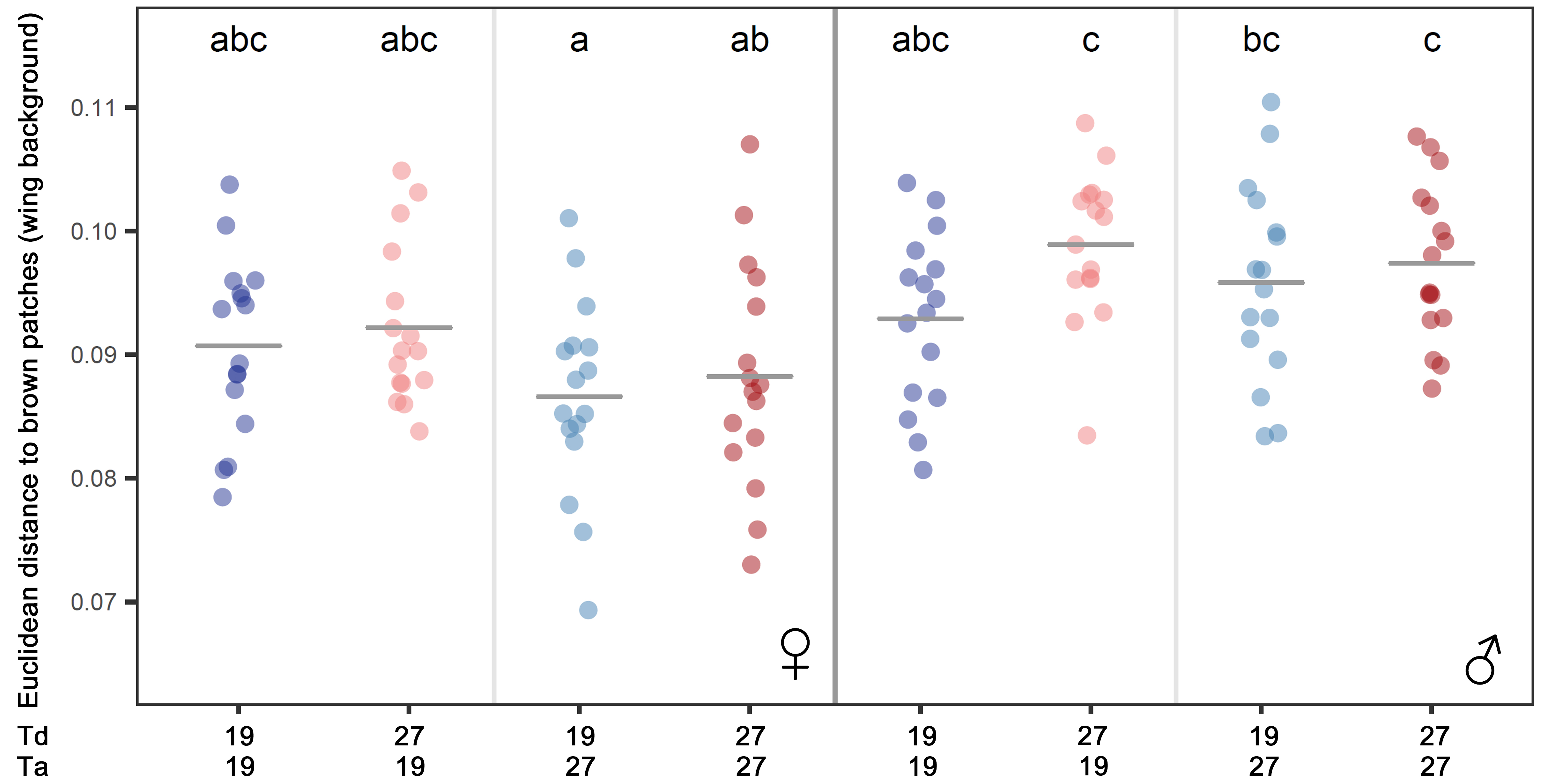 |  | F-value | df_KR_ | P_KR_ | | | |
|  |  | T_D_ | 3.79 | 1,56 | 0.057 | | | |
|  |  | T_A_ | 1.44 | 1,56 | 0.235 | | | |
|  |  | **Sex** | **24.72** | **1,56** | **<0.001** | | | |
|  |  | T_D_:T_A_ | 0.60 | 1,56 | 0.442 | | | |
|  |  | T_D_:Sex | 0.65 | 1,56 | 0.422 | | | |
|  |  | T_A_:Sex | 2.96 | 1,56 | 0.091 | | | |
|  |  | T_D_:T_A_:Sex | 0.69 | 1,56 | 0.410 | | | |
| *Continuation of Supplementary Table 1.* | | | | | |  |  |  |
| **C)** | 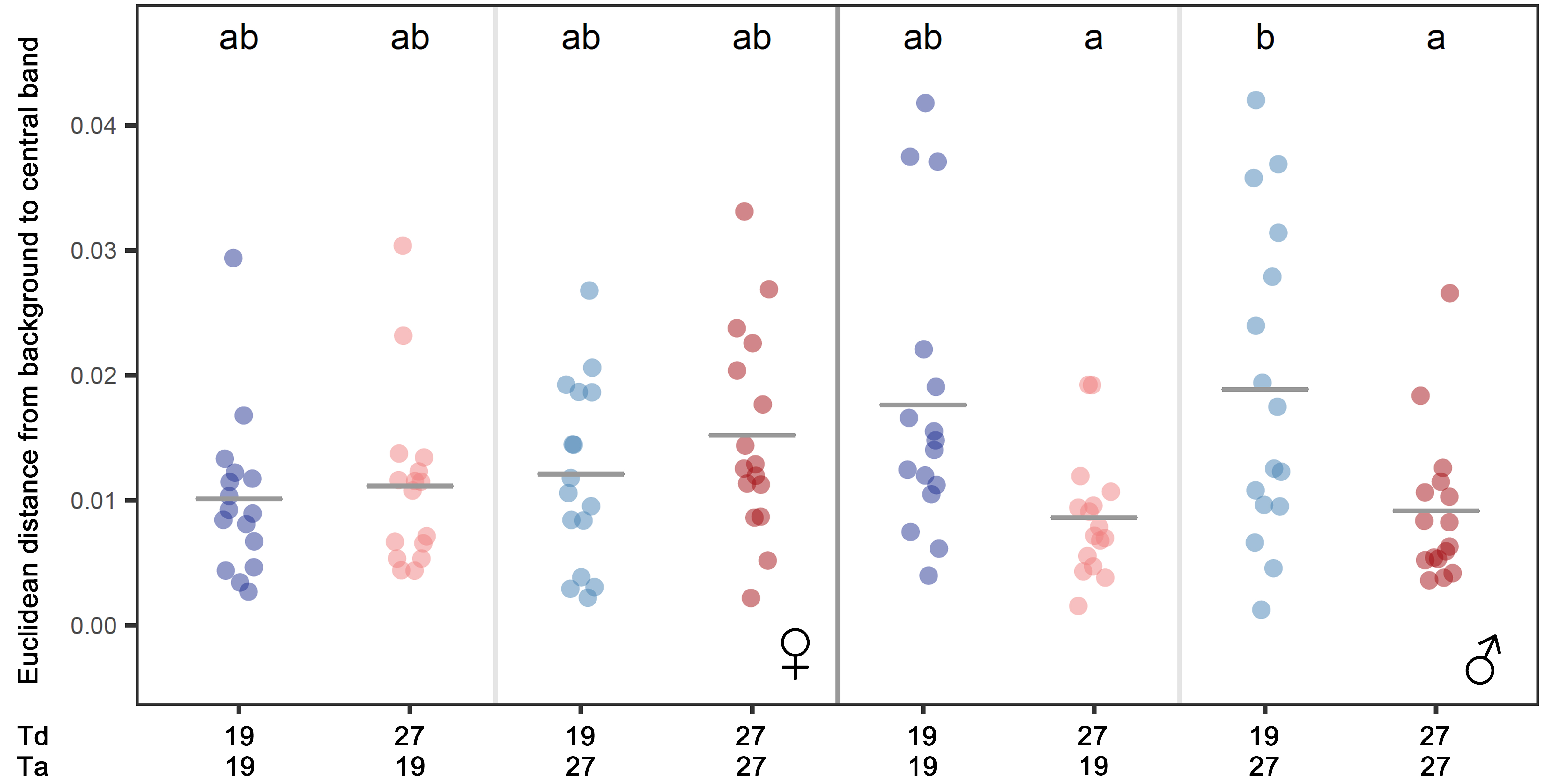 |  | F-value | df_KR_ | P_KR_ | | | |
|  |  | T_D_ | 5.86 | 1,56 | 0.019 | | | |
|  |  | T_A_ | 1.68 | 1,56 | 0.200 | | | |
|  |  | Sex | 0.89 | 1,56 | 0.349 | | | |
|  |  | T_D_:T_A_ | 0.05 | 1,56 | 0.821 | | | |
|  |  | **T_D_:Sex** | **14.38** | **1,56** | **<0.001** | | | |
|  |  | T_A_:Sex | 0.51 | 1,56 | 0.480 | | | |
|  |  | T_D_:T_A_:Sex | 0.22 | 1,56 | 0.644 | | | |
|  | | | | | |  |  |  |
| **D)** | 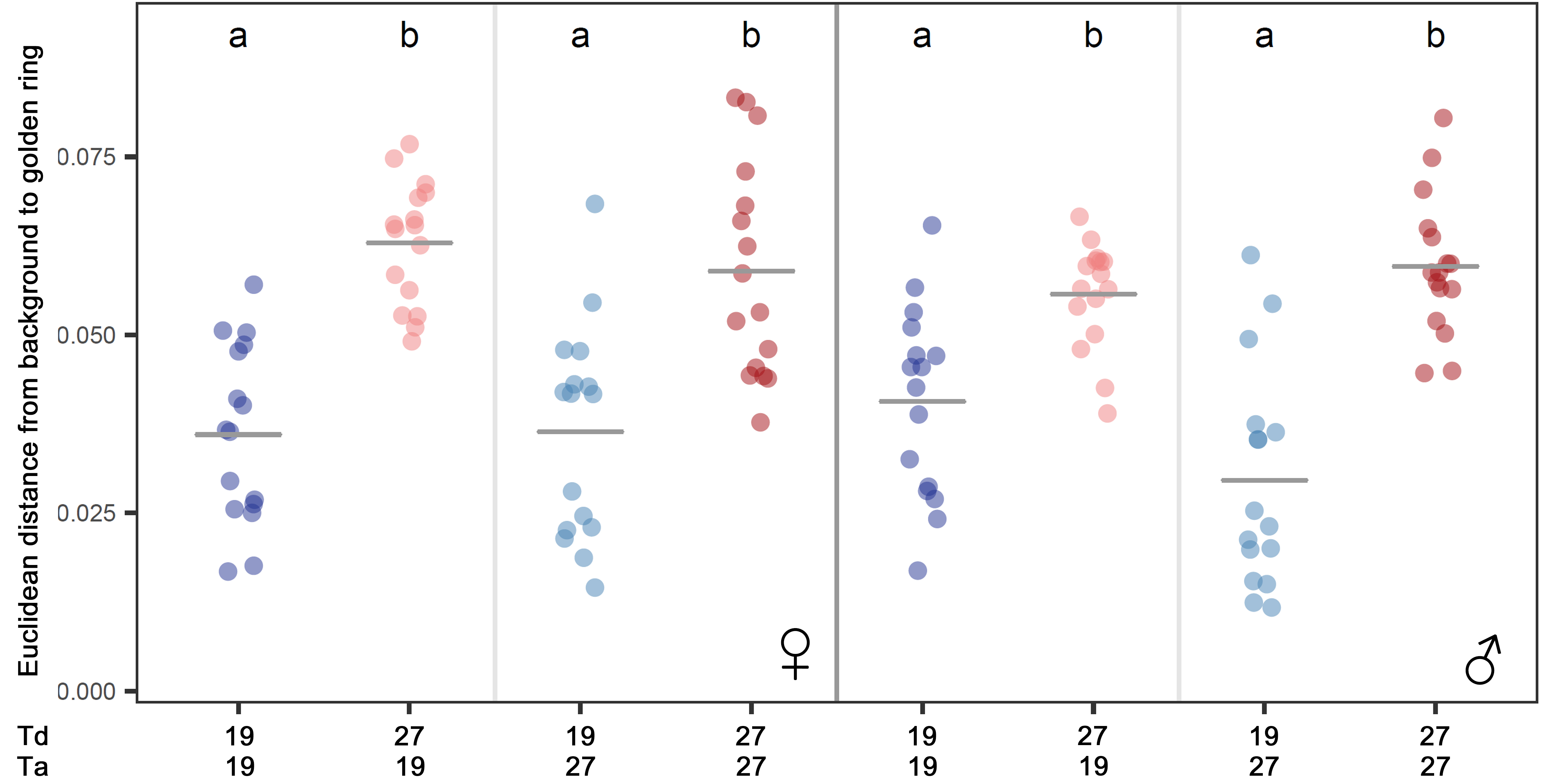 |  | F-value | df_KR_ | P_KR_ | | | |
|  |  | **T_D_** | **114.23** | **1,56** | **<0.001** | | | |
|  |  | T_A_ | 1.45 | 1,56 | 0.233 | | | |
|  |  | Sex | 0.97 | 1,56 | 0.328 | | | |
|  |  | T_D_:T_A_ | 1.43 | 1,56 | 0.236 | | | |
|  |  | T_D_:Sex | 0.24 | 1,56 | 0.623 | | | |
|  |  | T_A_:Sex | 0.16 | 1,56 | 0.686 | | | |
|  |  | T_D_:T_A_:Sex | 4.76 | 1,56 | 0.033 | | | |
|  | | | | | |  |  |  |
| **E)** | 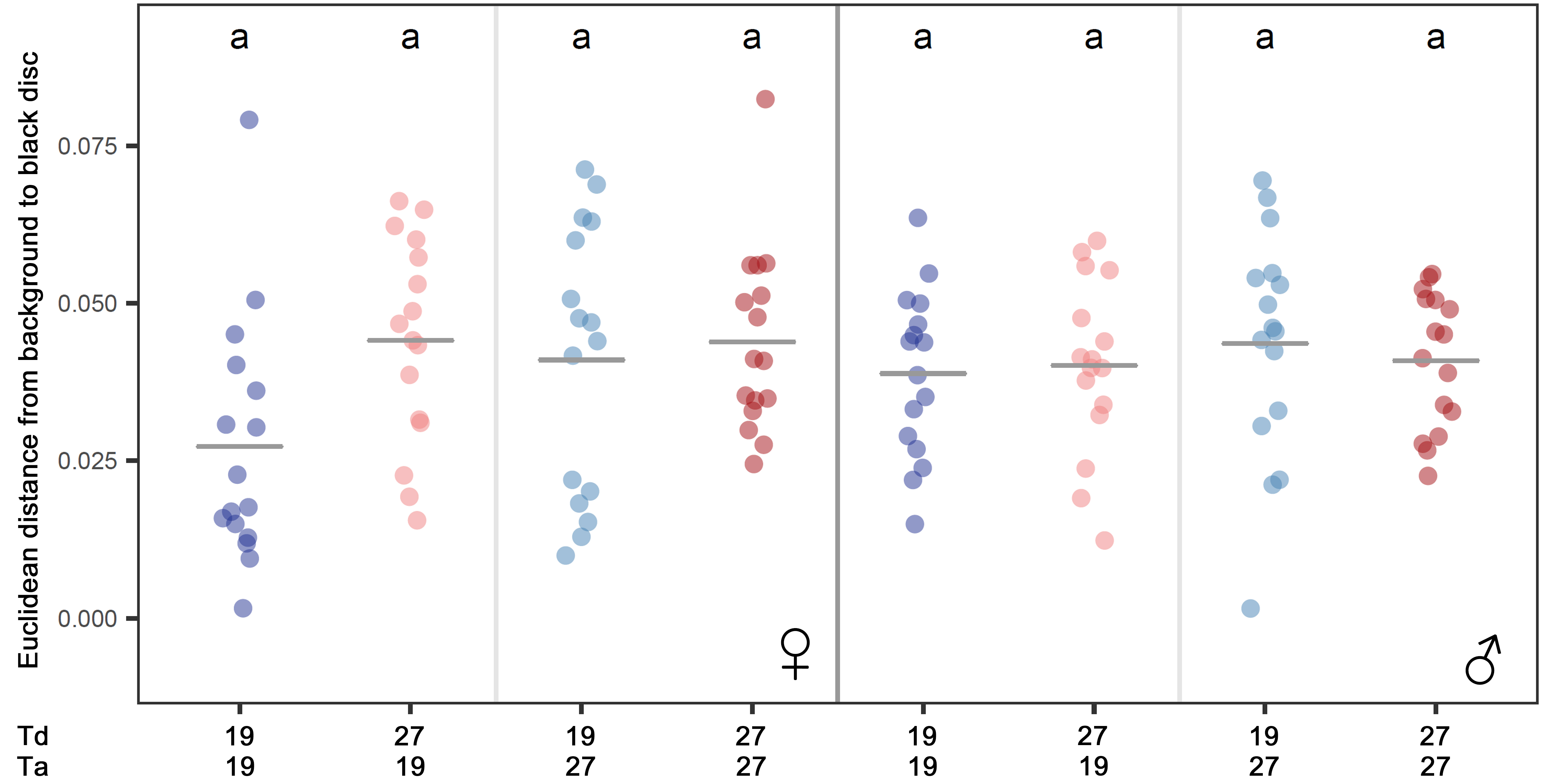 |  | F-value | df_KR_ | P_KR_ | | | |
|  |  | T_D_ | 2.05 | 1,56 | 0.158 | | | |
|  |  | T_A_ | 2.24 | 1,56 | 0.140 | | | |
|  |  | Sex | 0.33 | 1,56 | 0.571 | | | |
|  |  | T_D_:T_A_ | 1.98 | 1,56 | 0.165 | | | |
|  |  | T_D_:Sex | 2.74 | 1,56 | 0.104 | | | |
|  |  | T_A_:Sex | 0.39 | 1,56 | 0.535 | | | |
|  |  | T_D_:T_A_:Sex | 0.62 | 1,56 | 0.435 | | | |
|  | | | | | |  |  |  |
| **F)** | 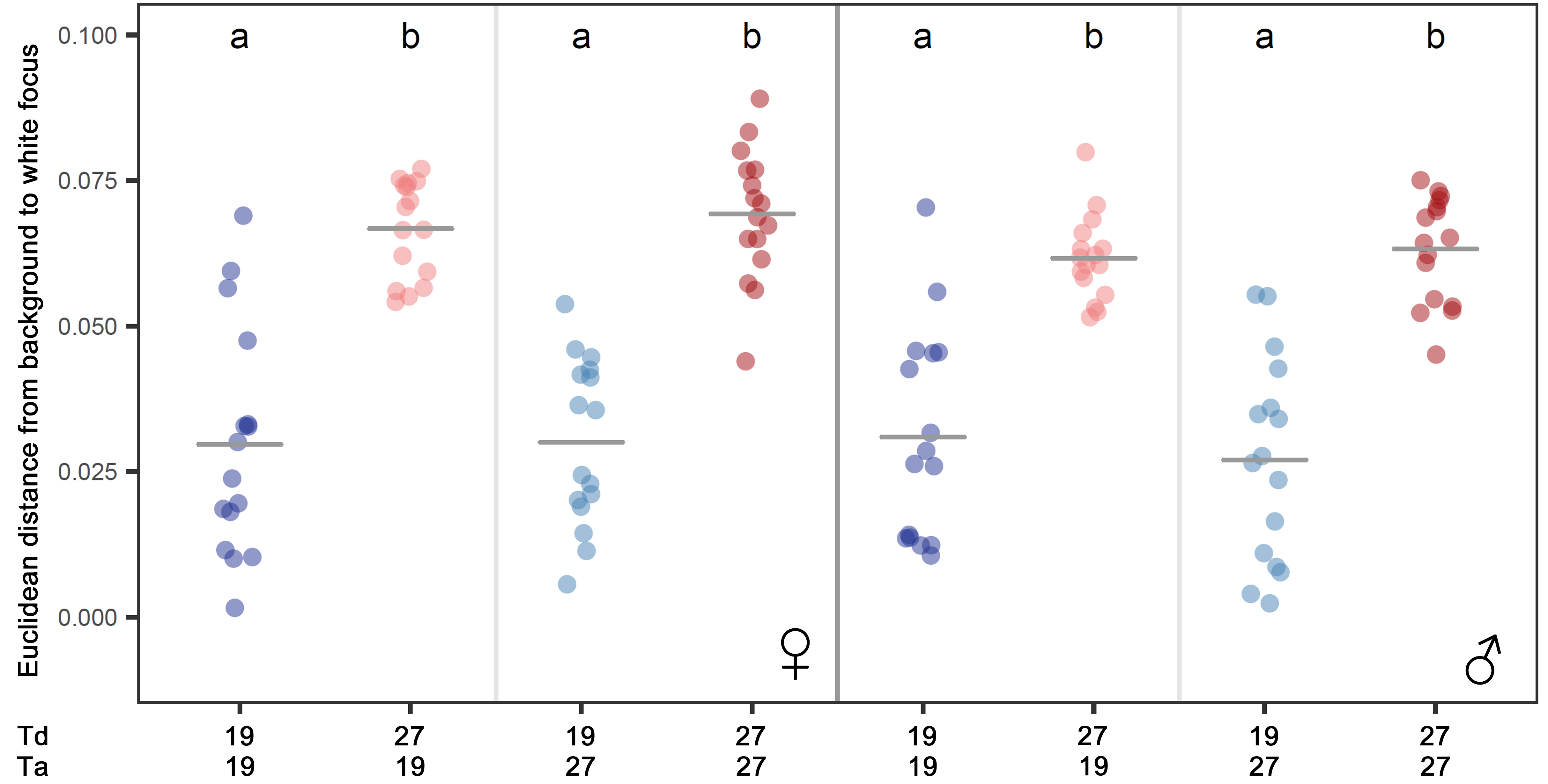 |  | F-value | df_KR_ | P_KR_ | | | |
|  |  | **T_D_** | **186.29** | **1,56** | **<0.001** | | | |
|  |  | T_A_ | <0.01 | 1,56 | 0.959 | | | |
|  |  | Sex | 1.51 | 1,56 | 0.224 | | | |
|  |  | T_D_:T_A_ | 0.52 | 1,56 | 0.474 | | | |
|  |  | T_D_:Sex | 0.79 | 1,56 | 0.377 | | | |
|  |  | T_A_:Sex | 0.25 | 1,56 | 0.622 | | | |
|  |  | T_D_:T_A_:Sex | 0.10 | 1,56 | 0.754 | | | |
| *Continuation of Supplementary Table 1.* | | | | | |  |  |  |
| **G)** | 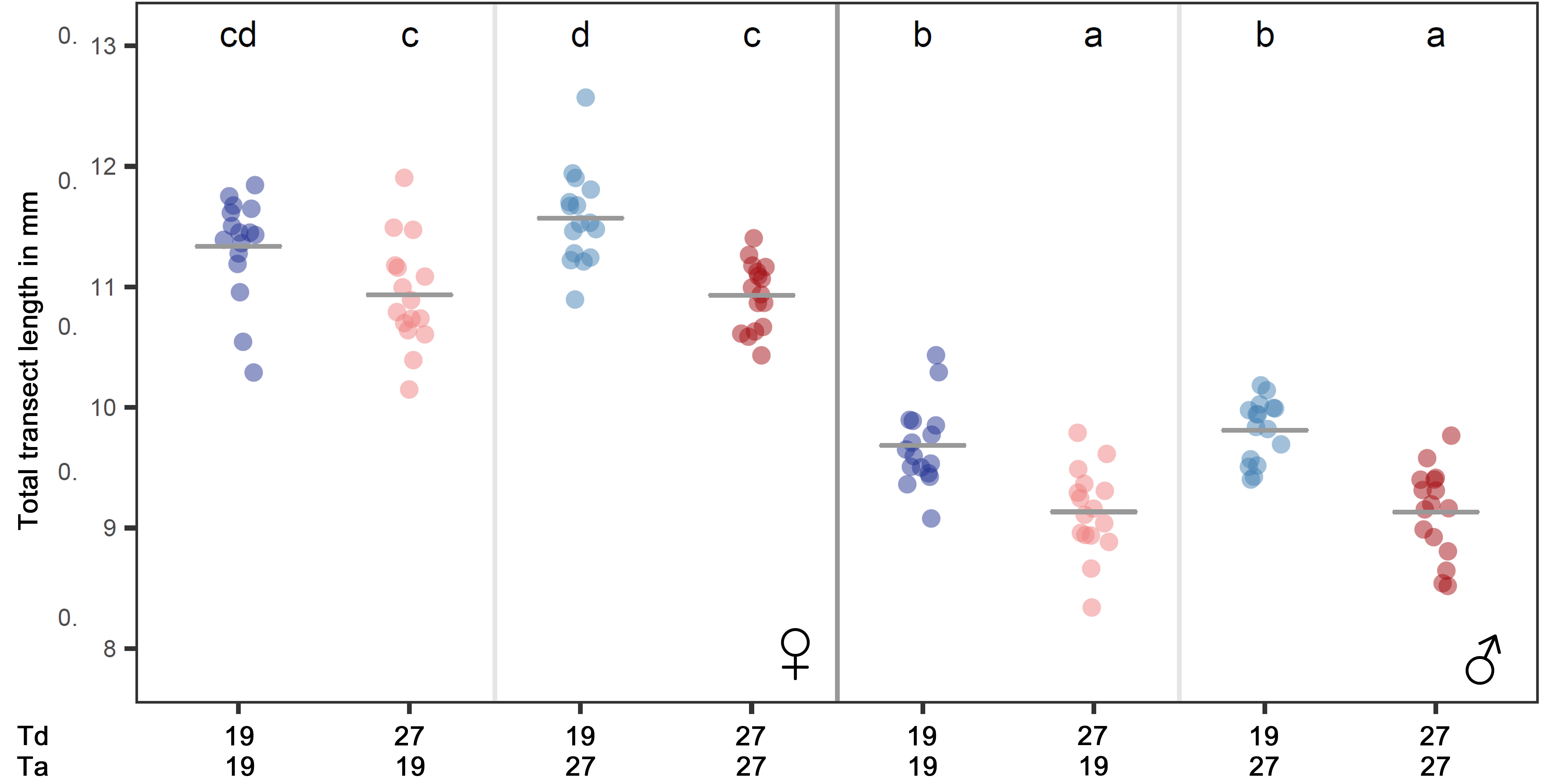 |  | F-value | df_KR_ | P_KR_ | | | |
|  |  | **T_D_** | **70.18** | **1,56** | **<0.001** | | | |
|  |  | T_A_ | 1.73 | 1,56 | 0.194 | | | |
|  |  | **Sex** | **668.04** | **1,56** | **<0.001** | | | |
|  |  | T_D_:T_A_ | 1.80 | 1,56 | 0.185 | | | |
|  |  | T_D_:Sex | 0.47 | 1,56 | 0.495 | | | |
|  |  | T_A_:Sex | 0.15 | 1,56 | 0.698 | | | |
|  |  | T_D_:T_A_:Sex | 0.16 | 1,56 | 0.690 | | | |
|  | | | | | |  |  |  |
| **H)** | 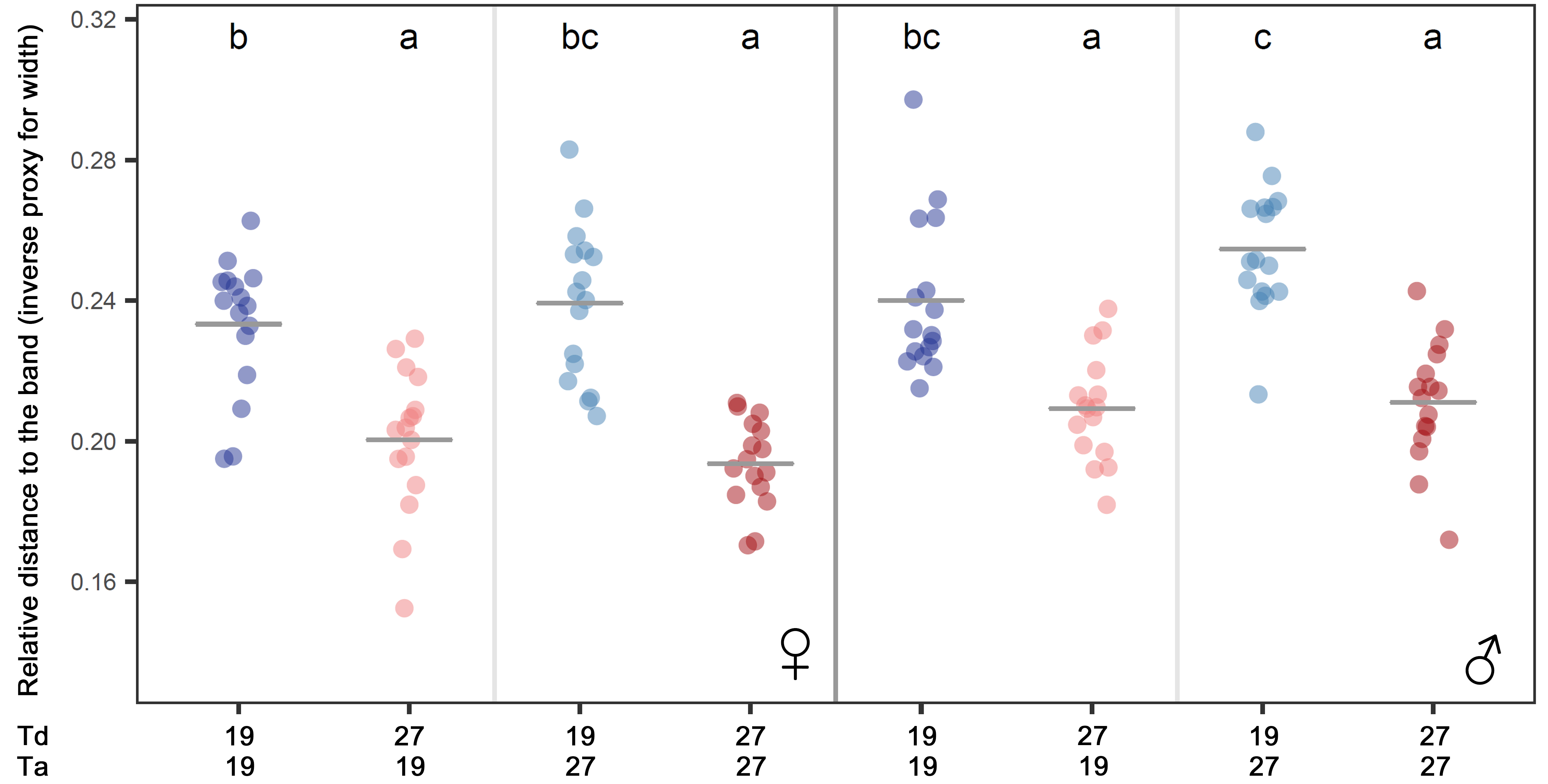 |  | F-value | df_KR_ | P_KR_ | | | |
|  |  | **T_D_** | **134.93** | **1,56** | **<0.001** | | | |
|  |  | T_A_ | 1.40 | 1,56 | 0.242 | | | |
|  |  | **Sex** | **13.55** | **1,56** | **<0.001** | | | |
|  |  | T_D_:T_A_ | 3.78 | 1,56 | 0.057 | | | |
|  |  | T_D_:Sex | 0.10 | 1,56 | 0.750 | | | |
|  |  | T_A_:Sex | 1.72 | 1,56 | 0.195 | | | |
|  |  | T_D_:T_A_:Sex | <0.01 | 1,56 | 0.990 | | | |
|  | | | | | |  |  |  |
| **I)** | 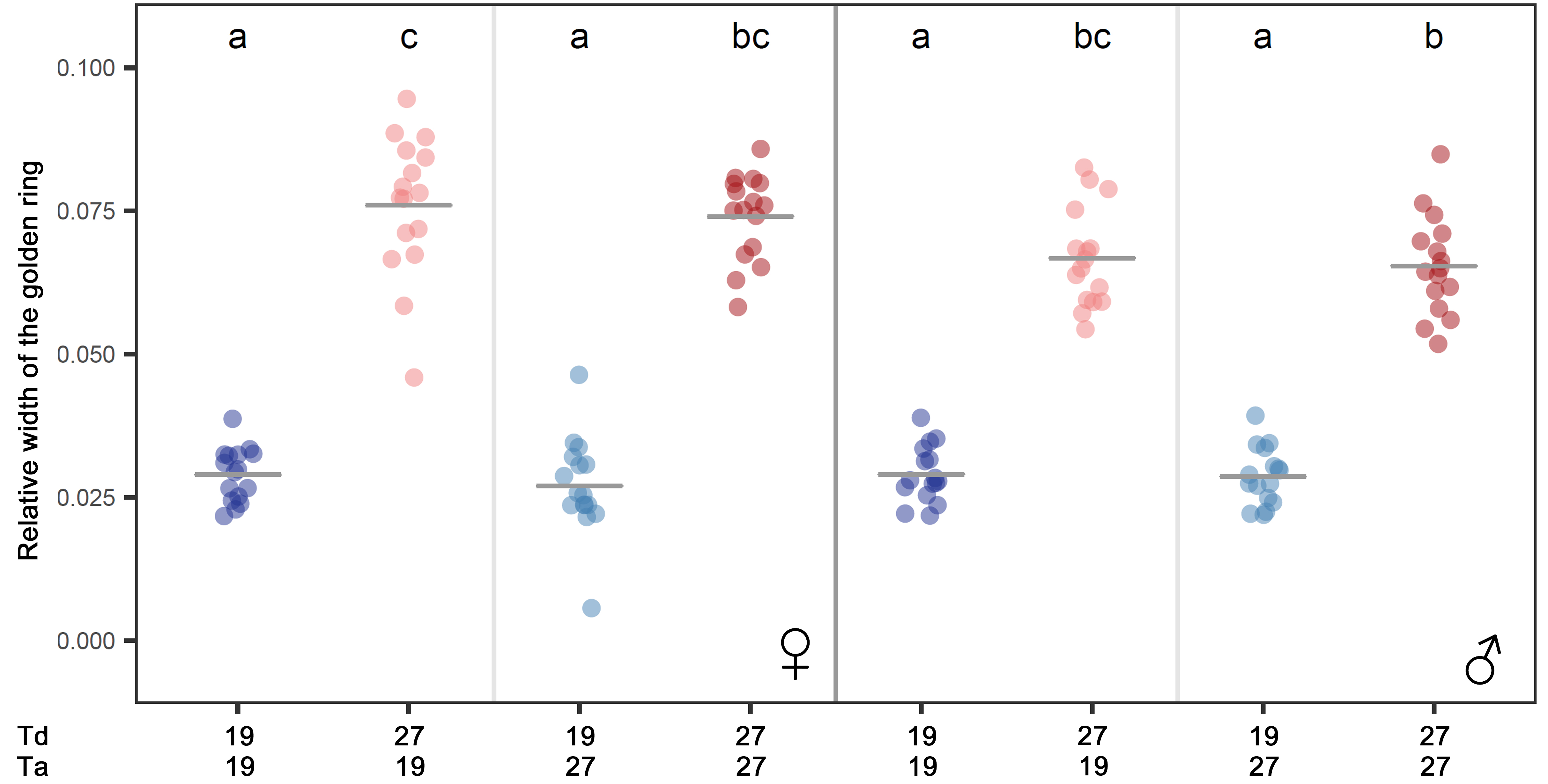 |  | F-value | df_KR_ | P_KR_ | | | |
|  |  | **T_D_** | **748.96** | **1,56** | **<0.001** | | | |
|  |  | T_A_ | 0.84 | 1,56 | 0.363 | | | |
|  |  | Sex | 6.89 | 1,56 | 0.011 | | | |
|  |  | T_D_:T_A_ | 0.02 | 1,56 | 0.878 | | | |
|  |  | **T_D_:Sex** | **10.05** | **1,56** | **0.002** | | | |
|  |  | T_A_:Sex | 0.13 | 1,56 | 0.723 | | | |
|  |  | T_D_:T_A_:Sex | 0.03 | 1,56 | 0.874 | | | |
|  | | | | | |  |  |  |
| **J)** | 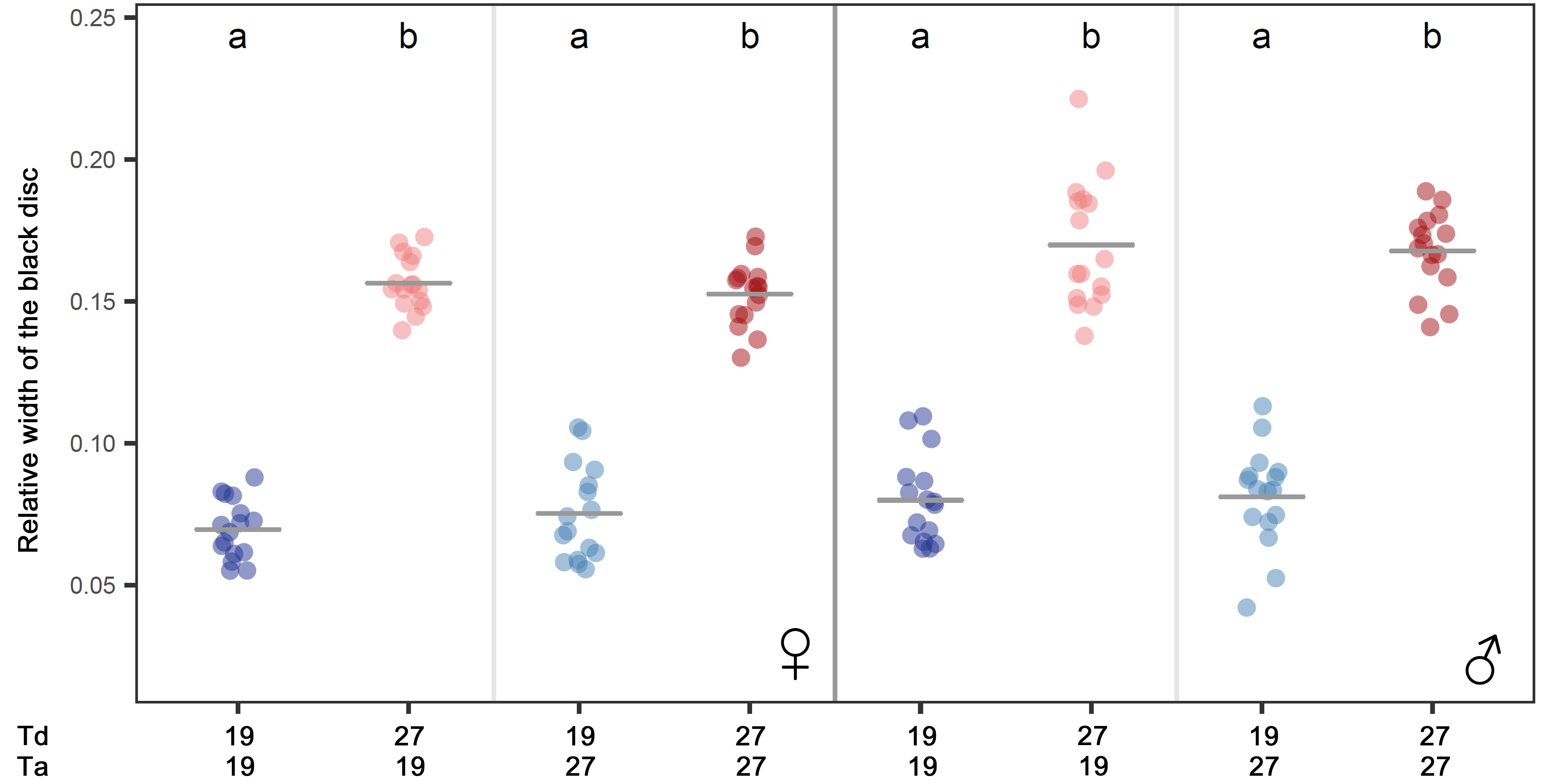 |  | F-value | df_KR_ | P_KR_ | | | |
|  |  | **T_D_** | **774.64** | **1,56** | **<0.001** | | | |
|  |  | T_A_ | <0.01 | 1,56 | 0.946 | | | |
|  |  | **Sex** | **13.36** | **1,56** | **<0.001** | | | |
|  |  | T_D_:T_A_ | 1.08 | 1,56 | 0.303 | | | |
|  |  | T_D_:Sex | 1.04 | 1,56 | 0.313 | | | |
|  |  | T_A_:Sex | 0.05 | 1,56 | 0.833 | | | |
|  |  | T_D_:T_A_:Sex | 0.26 | 1,56 | 0.614 | | | |
| *Continuation of Supplementary Table 1.* | | | | | |  |  |  |
| **K)** | 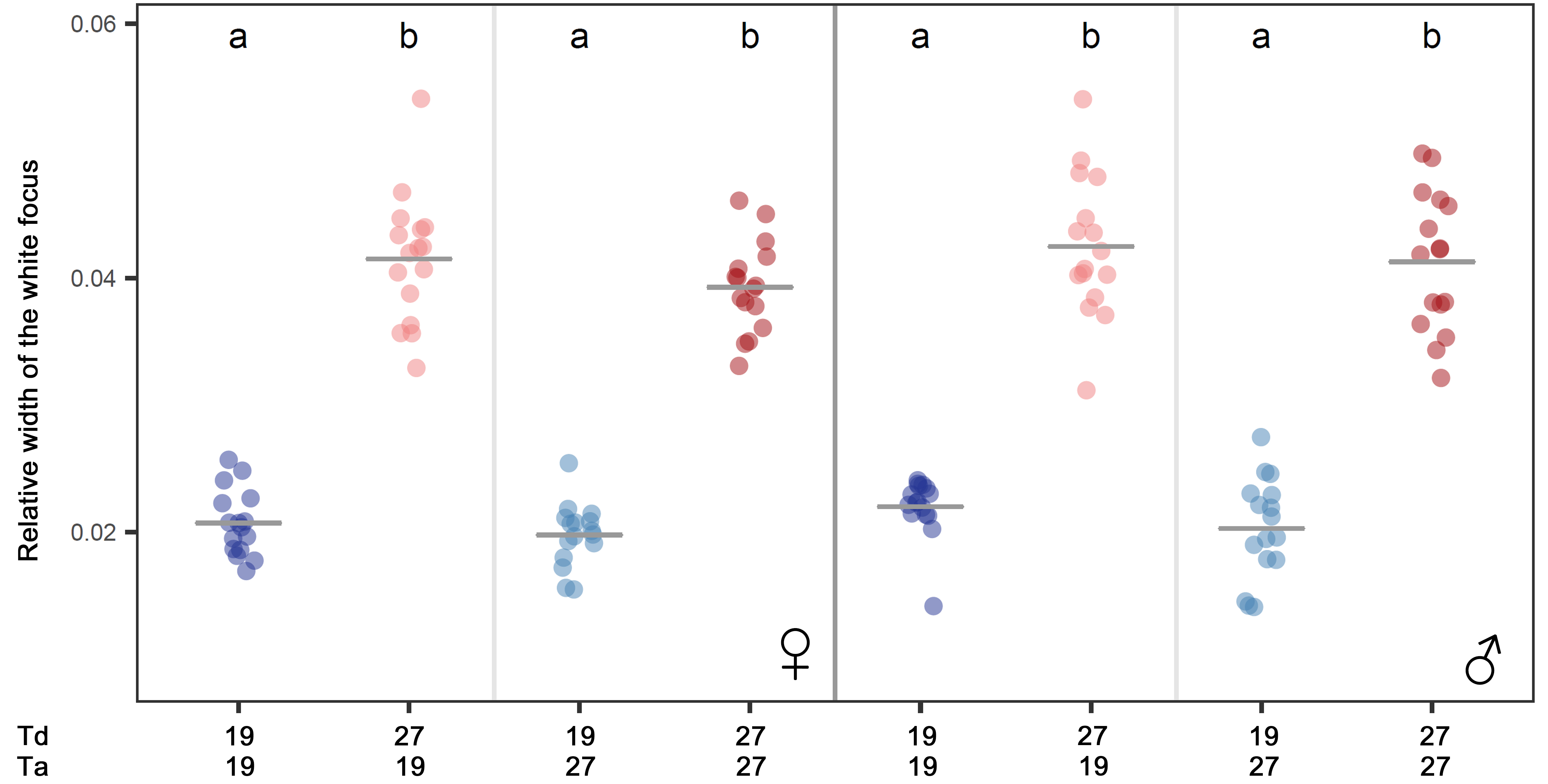 |  | F-value | df_KR_ | P_KR_ | | | |
|  |  | **T_D_** | **627.26** | **1,56** | **<0.001** | | | |
|  |  | T_A_ | 3.46 | 1,56 | 0.068 | | | |
|  |  | Sex | 2.14 | 1,56 | 0.149 | | | |
|  |  | T_D_:T_A_ | 0.06 | 1,56 | 0.815 | | | |
|  |  | T_D_:Sex | 0.13 | 1,56 | 0.719 | | | |
|  |  | T_A_:Sex | <0.01 | 1,56 | 0.938 | | | |
|  |  | T_D_:T_A_:Sex | 0.30 | 1,56 | 0.585 | | | |

| **Supplementary Table 2:** GLMM for perching preference (related to Fig 2a in the main text). **A.** Minimum adequate models were obtained by backward elimination, starting from the full model and using Bayesian Information Criterion (BIC). **B.** P-values for the minimum adequate model were calculated via likelihood ratio tests and parametric bootstrap (nsim=999), using the package *afex*. **C.** Mean probabilities, as well as upper and lower confidence limits, are given for each sex and experimental treatment. Post-hoc pairwise comparisons (alpha=0.05), obtained using the package *lsmeans*, specify differences between experimental treatments and sexes. | | | | | | | | | | | | | | | | |  |  |
| --- | --- | --- | --- | --- | --- | --- | --- | --- | --- | --- | --- | --- | --- | --- | --- | --- | --- | --- |
| **A.** Perching preference: cbind(newBrown,newGreen) | | | | | | | | | | | | | | | BIC | | | |
| M_full_ : | | T_D_ * T_A_ * Sex | | | | | | + (1|Cohort) + (1|Timepoint) | | | | | | 2846.96 | | | | |
| M_2_ : | | T_D_ + T_A_ + Sex + T_D_:T_A_ + T_D_:Sex + T_A_:Sex | | | | | | + (1|Cohort) + (1|Timepoint) | | | | | | 2842.95 | | | | |
| M_3_ : | | T_D_ + T_A_ + Sex + T_D_:Sex + T_A_:Sex | | | | | | + (1|Cohort) + (1|Timepoint) | | | | | | 2836.78 | | | | |
| **M_mam_ :** | | **T_D_ + T_A_ + Sex + T_A_:Sex** | | | | | | **+ (1|Cohort) + (1|Timepoint)** | | | | | | **2829.95** | | | | |
| M_5_ : | | T_D_ + T_A_ + Sex | | | | | | + (1|Cohort) + (1|Timepoint) | | | | | | 2837.34 | | | | |
|  | | | | | | | | | | | | | | | | |  |  |
| **B.** | Chisq | | P_LRT_ | P_PB (n=999)_ |  | **C.** | Sex | | T_A_ | T_D_ | mean | LCL | UCL | | | pairs | | |
| **T_D_** | **20.66** | | **<0.001** | **0.001** |  |  | F | | 19 | DS | 0.624 | 0.575 | 0.671 | | | E | | |
| T_A_ | 1.32 | | 0.251 | 0.245 |  |  | F | | 19 | WS | 0.566 | 0.518 | 0.613 | | | CD | | |
| **Sex** | **62.53** | | **<0.001** | **0.001** |  |  | F | | 27 | DS | 0.590 | 0.553 | 0.627 | | | DE | | |
| **T_A_:Sex** | **14.26** | | **<0.001** | **0.001** |  |  | F | | 27 | WS | 0.530 | 0.493 | 0.566 | | | C | | |
|  |  | |  |  |  |  | M | | 19 | DS | 0.448 | 0.408 | 0.490 | | | B | | |
|  |  | |  |  |  |  | M | | 19 | WS | 0.389 | 0.349 | 0.431 | | | A | | |
|  |  | |  |  |  |  | M | | 27 | DS | 0.516 | 0.479 | 0.552 | | | C | | |
|  |  | |  |  |  |  | M | | 27 | WS | 0.455 | 0.419 | 0.492 | | | B | | |

| **Supplementary Table 3****:** GLMM for activity levels (related to Fig 2b in the main text). **A.** Minimum adequate models were obtained by backward elimination, starting from the full model and using Bayesian Information Criterion (BIC). **B.** P-values for the minimum adequate model were calculated via likelihood ratio tests and parametric bootstrap (nsim=999), using the package *afex*. **C.** Mean probabilities, as well as upper and lower confidence limits, are given for each sex and experimental treatment. Post-hoc pairwise comparisons (alpha=0.05), obtained using the package *lsmeans*, specify differences between experimental treatments and sexes. | | | | | | | | | | | | | | | | |  |  |
| --- | --- | --- | --- | --- | --- | --- | --- | --- | --- | --- | --- | --- | --- | --- | --- | --- | --- | --- |
| **A.** Activity level: cbind(leaving,staying) | | | | | | | | | | | | | | | BIC | | | |
| M_full_ : | | T_D_ * T_A_ * Sex | | | | | | + (1|Cohort) + (1|Timepoint) | | | | | | 4362.39 | | | | |
| M_2_ : | | T_D_ + T_A_ + Sex + T_D_:T_A_ + T_D_:Sex + T_A_:Sex | | | | | | + (1|Cohort) + (1|Timepoint) | | | | | | 4360.09 | | | | |
| **M**_mam_**:** | | **T_D_ + T_A_ + Sex + T_D_:Sex + T_A_:Sex** | | | | | | **+ (1|Cohort) + (1|Timepoint)** | | | | | | **4354.53** | | | | |
| M_4_ : | | T_D_ + T_A_ + Sex + T_A_:Sex | | | | | | + (1|Cohort) + (1|Timepoint) | | | | | | 4355.46 | | | | |
| M_5_ : | | T_D_ + T_A_ + Sex + T_D_:Sex | | | | | | + (1|Cohort) + (1|Timepoint) | | | | | | 4370.57 | | | | |
|  | | | | | | | | | | | | | | | | |  |  |
| **B.** | Chisq | | P_LRT_ | P_PB (n=999)_ |  | **C.** | Sex | | T_A_ | T_D_ | mean | LCL | UCL | | | pairs | | |
| **T_D_** | **8.54** | | **<0.001** | **0.006** |  |  | F | | 19 | DS | 0.554 | 0.472 | 0.634 | | | B | | |
| **T_A_** | **13.14** | | **<0.001** | **0.001** |  |  | F | | 19 | WS | 0.551 | 0.470 | 0.629 | | | B | | |
| Sex | 0.25 | | 0.620 | 0.661 |  |  | F | | 27 | DS | 0.588 | 0.511 | 0.661 | | | B | | |
| **T_D_:Sex** | **7.79** | | **0.005** | **0.013** |  |  | F | | 27 | WS | 0.585 | 0.508 | 0.657 | | | B | | |
| **T_A_:Sex** | **22.91** | | **<0.001** | **0.001** |  |  | M | | 19 | DS | 0.738 | 0.669 | 0.797 | | | C | | |
|  |  | |  |  |  |  | M | | 19 | WS | 0.617 | 0.540 | 0.689 | | | B | | |
|  |  | |  |  |  |  | M | | 27 | DS | 0.544 | 0.467 | 0.619 | | | B | | |
|  |  | |  |  |  |  | M | | 27 | WS | 0.406 | 0.333 | 0.482 | | | A | | |
